# MicroRNA profiling associated with muscle growth in modern broilers compared to an unselected chicken breed

**DOI:** 10.1186/s12864-018-5061-7

**Published:** 2018-09-17

**Authors:** Bhuwan Khatri, Dongwon Seo, Stephanie Shouse, Jeong Hoon Pan, Nicholas J. Hudson, Jae Kyeom Kim, Walter Bottje, Byungwhi C. Kong

**Affiliations:** 1 Department of Poultry Science, Center of Excellence for Poultry Science, University of Arkansas, Fayetteville, AR 72701 USA; 2 School of Human Environmental Sciences, University of Arkansas, Fayetteville, AR 72701 USA; 3 School of Agriculture and Food Sciences, The University of Queensland, QLD4343, Gatton, Australia

**Keywords:** miRNA, Pedigree male broiler, Barred Plymouth Rock, Production efficiency, Differential expression

## Abstract

**Background:**

Genetically selected modern broiler chickens have acquired outstanding production efficiency through rapid growth and improved feed efficiency compared to unselected chicken breeds. Recently, we analyzed the transcriptome of breast muscle tissues obtained from modern pedigree male (PeM) broilers (rapid growth and higher efficiency) and foundational Barred Plymouth Rock (BPR) chickens (slow growth and poorer efficiency). This study was designed to investigate microRNAs that play role in rapid growth of the breast muscles in modern broiler chickens.

**Results:**

In this study, differential abundance of microRNA (miRNA) was analyzed in breast muscle of PeM and BPR chickens and the results were integrated with differentially expressed (DE) mRNA in the same tissues. A total of 994 miRNA were identified in PeM and BPR chicken lines from the initial analysis of small RNA sequencing data. After filtering and statistical analyses, the results showed miR-2131-5p, miR-221-5p, miR-126-3p, miR-146b-5p, miR-10a-5p, let-7b, miR-125b-5p, and miR-146c-5p up-regulated whereas miR-206 down-regulated in PeM compared to BPR breast muscle. Based on inhibitory regulations of miRNAs on the mRNA abundance, our computational analysis using miRDB, an online software, predicated that 118 down-regulated mRNAs may be targeted by the up-regulated miRNAs, while 35 up-regulated mRNAs appear to be due to a down-regulated miRNA (i.e., miR-206). Functional network analyses of target genes of DE miRNAs showed their involvement in calcium signaling, axonal guidance signaling, and NRF2-mediated oxidative stress response pathways suggesting their involvement in breast muscle growth in chickens.

**Conclusion:**

From the integrated analyses of differentially expressed miRNA-mRNA data, we were able to identify breast muscle specific miRNAs and their target genes whose concerted actions can contribute to rapid growth and higher feed efficiency in modern broiler chickens. This study provides foundation data for elucidating molecular mechanisms that govern muscle growth in chickens.

**Electronic supplementary material:**

The online version of this article (10.1186/s12864-018-5061-7) contains supplementary material, which is available to authorized users.

## Background

Genetically selected modern broiler chickens are characterized by rapid growth and improved feed efficiency compared to unselected counterparts. These traits are beneficial to meet the global protein needs for an ever increasing human population [1, 2]. Understanding the mechanism behind rapid muscle growth and high feed efficiency in chickens will help in maintaining sustainable protein source through improved animal production system.

MicroRNAs are short 18–24 nucleotides long non-coding regulatory RNAs that target mRNAs for cleavage, deadenylation or translational inhibition of gene expression at post-transcriptional level [3, 4]. In recent years, many studies have shown the vital roles of miRNAs in various aspects of biological phenomenon associated with growth and development [5–8]. Recently, a total of 921 miRNAs were identified from breast muscles of fast and slow growing broilers [9]. Of note, let-7b was experimentally validated through genetic analyses in chickens to affect signaling pathways regulating skeletal muscle growth [10]. The miR-1 was shown to promote myogenesis by targeting histone deacetylase (HDAC) 4, a transcriptional repressor protein of muscle gene expression. The miR-133 was proven to enhance myoblast proliferation by repressing serum response factor (SRF) [11]. The miR-26a was reported to accelerate the process of myogenesis through induction of creatine kinase and up-regulation of *myoD* and *myogenin* [12]. All these reports suggest that miRNAs are important regulators of muscle growth and development in vertebrate animals.

Extensive genetic selection has led to rapid growth rate and large muscle mass in modern broilers compared to unselected chicken breeds [13]. In a previous study, we identified differentially expressed genes associated with breast muscle myogenesis in pedigree male (PeM) broilers (rapidly growing, higher efficiency, and large muscle mass) compared with Barred Plymouth Rock (BPR) chickens (slowly growing, poorer efficiency, and small muscle mass) [2]. This transcriptomic analysis indicated that rapid growth and large muscle mass shown in modern broilers may be due to altered mitochondrial functions, growth signaling pathways, oxidative stress pathway, and/or hormone receptor pathways. To elucidate regulatory roles of miRNAs on muscle growth and production efficiency, we profiled differentially expressed (DE) miRNAs using small RNA sequencing followed by prediction of potential target mRNAs; and eventually, miRNA profiling results were integrated with transcriptomic data of DE genes obtained by previous mRNA sequencing study [2].

## Methods

### Ethics statement

The present study was conducted in accordance with the recommendations in the guide for the care and use of laboratory animals of the National Institutes of Health. All procedures for animal care complied with the University of Arkansas Institutional Animal Care and Use Committee (IACUC): Protocol #14012.

### Samples

This study was conducted with modern pedigree male (PeM) broilers provided by Cobb Vantress, Inc. (Siloam Springs, AR) [14] and foundational Barred Plymouth Rock (BPR) chickens maintained at the Poultry Research Facilities of Arkansas Agricultural Experiment Station, University of Arkansas (Fayetteville, AR). Breast muscle tissues were obtained from PeM, highly selected for growth and feed efficiency [14, 15], and BPR as described elsewhere [2]. Briefly, immature PeM and BPR chickens (≤8 weeks old, *n* = 6 per breed) were killed by an overdose of sodium pentobarbital (i.v. injection) and breast muscle tissue was obtained and flash frozen in liquid nitrogen. Total RNAs were extracted from the muscle tissue using TRIzol reagent (Thermo-Fisher Scientific, Carlsbad, CA) following manufacturer’s protocol. Extracted RNA samples were treated with DNase I (New England Biolabs Inc., Ipswich, MA) and purified again using TRIzol reagent. RNA quality was then assessed using Agilent 2200 TapeStation instrument (Santa Clara, CA). All RNA samples showed high enough quality and quantity (RNA Integrity Number; RIN > 5.5; data not shown), that were confirmed by sequencing core facility.

### MicroRNA sequencing and data analysis

Library preparation for individual samples and sequencing were performed by the Research Technology Support Facility at Michigan State University (East Lansing, MI). Illumina TruSeq system 1 × 50 bp single end read method was used for miRNA sequencing. Quality of raw reads were determined using a FastQC tool kit [16] and adapters were trimmed using bbduk.sh command line of BBMap toolkit (https://sourceforge.net/projects/bbmap/). The clean reads were then aligned to reference mature miRNA sequences of *Gallus gallus* obtained from miRBase (http://mirbase.org/) using the Arraystar program in Lasergene software package (DNAStar, Madison, WI) and read counts were normalized by reads per millions to stabilize the variance. Differential expression with normalized read counts was further analyzed using JMP Genomics 9 (SAS Institute Inc., Cary, NC). MicroRNAs with less than 5 average read counts in both comparison groups were not considered for further analysis. The t-statistics was used to compare abundances between PeM and BPR, and miRNAs showing fold change > 1.2 and *p*-value < 0.05 were considered as DE.

### Hierarchical clustering

DE miRNAs of PeM and BPR were subjected to hierarchical cluster analysis using JMP Genomics Program. A matrix with as many columns as birds (12) and as many rows as DE miRNA (9) were imported in which each cell contained log2 transformed fold change value for that miRNA and bird into JMP Genomics Program, normalizing on rows. After, hierarchical clustering on both rows and columns were applied followed by dendrogram image production.

### Target prediction of DE miRNA

Online miRNA target prediction tool, miRDB (http://www.mirdb.org/) was used to predict potential targets of DE miRNAs. The predicted targets of DE miRNA were then integrated with DE mRNA list obtained from same breast muscle tissue in our earlier study [2]. The mRNA showing opposite direction of expression to their corresponding miRNA were chosen as targets of DE miRNAs and used for subsequent bioinformatics analyses.

### Ingenuity pathway analysis

Ingenuity Pathway Analysis (IPA; Qiagen, Valencia, CA; https://www.qiagenbioinformatics.com/) software was used for construction of interaction network between DE miRNA and their candidate targets. Since IPA is based on bioinformatics in humans, functionalities for DE miRNAs in the chicken datasets are principally based upon mammalian biological mechanisms. As investment in biomedical research biases the functional annotations towards human disease, we have attempted to draw plausible conclusions based on avian literature [2]. All target genes of DE miRNAs were subjected to IPA analysis for functional annotation and canonical pathways mapping among which all target genes were identified by IPA.

### Small RNA purification, cDNA synthesis and quantitative real time PCR (qPCR)

Sixty micrograms of total RNA samples from 6 muscle samples each for PeM and BPR were used for small RNA enrichment and subsequent validation of miRNA sequencing results by qPCR. Small RNAs were enriched using mirVana miRNA isolation kit (Ambion, Carlsbad, CA) following manufacturer’s instructions. Enriched small RNAs were polyadenylated using Poly(A) Polymerase (Ambion) and re-purified using QIAquick Nucleotide Removal Kit (Qiagen). The polyadenylated small RNAs were then ligated with RNA oligonucleotide adapter (Table 1), treated with RNaseOUT and reverse transcribed to cDNA using adapter primer and SuperScript III reverse transcriptase (Thermo-Fisher Scientific). The cDNA samples were diluted to 1:10 ratio and a portion (2 μl) of cDNA was used for qPCR reaction using ABI prism 7500HT system (Thermo-Fisher Scientific) with PowerUp SYBR Green Master Mix (Thermo-Fisher Scientific). Primers were synthesized by Integrated DNA Technologies (Coralville, IA), and are listed in Table 1. The qPCR condition was as follows: 1 cycle at 95 °C for 2 min, 40 cycles at 95 °C for 30 s, 60 °C for 30 s. The chicken 5S ribosomal RNA was used as internal control. Dissociation curves were generated at the end of amplification process for validating data quality. All qPCR reactions were conducted three times and values of average cycle threshold (Ct) were determined for each sample, and 2^-ΔΔCt^ values for the comparison of PeM and BPR were used for relative quantification by fold-change and statistical significance.

**Table 1:** Primers used for qPCR. The first column indicates primer names and the second column shows their sequences

Name	Sequence
RTQ_primer	CGAATTCTAGAGCTCGAGGCAGGCGACATGGCTGGCTAGTTAAGCTTG GTACCGAGCTCGGATCCACTAGTCCTTTTTTTTTTTTTTTTTTTTTTTTTVN
RTQ-UNIr	CGAATTCTAGAGCTCGAGGCAGG
miR-146c-5p	TGAGAACTGAATTCCATGGACTG
miR-146b-5p	TGAGAACTGAATTCCATAGGCG
miR-10a-5p	TACCCTGTAGATCCGAATTTGT
miR-2131-5p	CTGTTACTGTTCTTCTGATGG
miR-221-5p	AACCTGGCATACAATGTAGATTTCTGT
miR-10b-5p	TACCCTGTAGAACCGAATTTGT
let-7b	TGAGGTAGTAGGTTGTGTGGTT
miR-125b-5p	TCCCTGAGACCCTAACTTGTGA
miR-206	TGGAATGTAAGGAAGTGTGTGG
5S_rRNA-F1	AAGCCTACAGCACCCGGTAT

## Results

### MicroRNA profiling in PeM and BPR chickens by miRNA sequencing

MicroRNA Sequencing of 12 samples yielded 33,727,148 and 51,310,328 raw sequence reads from PeM and BPR samples, respectively. After adapter trimming, 17,751,585 and 22,922,027 clean reads remained in PeM and BPR, respectively (data not shown). After alignment of clean reads to chicken reference mature miRNA collections, a total of 994 mature miRNAs were identified in both PeM and BPR. Rarely expressed mature miRNAs (i.e., raw read count < 5) were filtered out, resulting in 38 miRNAs remained as meaningfully expressed and were used for subsequent analyses (Additional file 1).

### Differentially expressed miRNAs in PeM compared to BPR

**Fig. 1:**
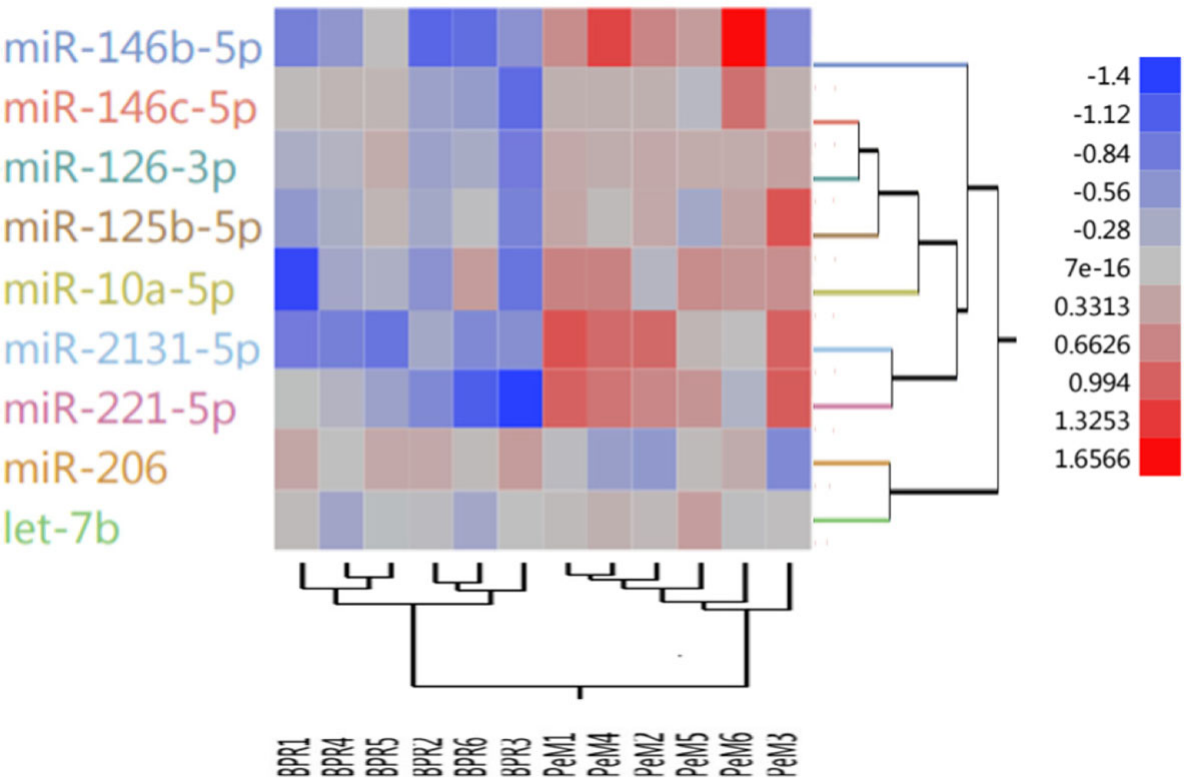
Hierarchically clustered heat map of 9 DE miRNA. Red and blue represent up and down-regulated expression in PeM respectively. Color density indicated level of fold change

Nine DE miRNAs showing *p*-value < 0.05 and fold change > 1.2 were identified in PeM compared with BPR (Table 2). Among 9 DE miRNAs, 8 miRNAs including miR-2131-5p, miR-221-5p, miR-126-3p, miR-146b-5p, miR-10a-5p, let-7b, miR-125b-5p, and miR-146c-5p were up-regulated while miR-206 was down-regulated in PeM compared to BPR breast muscle (Table 2). All DE miRNAs were validated using qPCR (Table 2). Our qPCR results indicated that expression patterns of 8 out of 9 miRNAs were in good agreement with miRNA sequencing data in terms of their direction and magnitude of change. One (miR-126-3p) out of 9 miRNAs did not match with miRNA sequencing which may be due to different approaches for data normalization. Additionally, hierarchical clustering showed clear discrimination of 12 birds into correct group of origin (Fig. 1).

**Table 2:** Comparison of fold change between miRNAseq and qPCR in breast muscle tissue of PeM compared to BPR broilers

miRNA	Sequence	miRNAseq		qPCR
		FC^a^	*p*-value	FC ± SEM^b^
miR-2131-5p	AUGCAGAAGUGCACGGAAACAGCU	2.62	5.71E-05	1.45 ± 0.24
miR-221-5p	AACCUGGCAUACAAUGUAGAUUUCUGU	2.42	0.001	1.43 ± 0.31
miR-126-3p^c^	UCGUACCGUGAGUAAUAAUGCGC	1.49	0.002	−0.87 ± 0.15^c^
miR-146b-5p	UGAGAACUGAAUUCCAUAGGCG	2.44	0.005	1.88 ± 0.30
miR-10a-5p	UACCCUGUAGAUCCGAAUUUGU	1.96	0.005	1.17 ± 0.10
miR-206	UGGAAUGUAAGGAAGUGUGUGG	−0.72	0.017	−0.62 ± 0.11
let-7b	UGAGGUAGUAGGUUGUGUGGUU	1.20	0.027	1.20 ± 0.16
miR-125b-5p	UCCCUGAGACCCUAACUUGUGA	1.51	0.028	1.17 ± 0.09
miR-146c-5p	UGAGAACUGAAUUCCAUGGACUG	1.42	0.049	1.31 ± 0.13

^a^Values denote linear fold changes obtained from miRNAseq analyses

^b^Values denote linear fold changes plus/minus standard error of the mean (SEM) from qPCR

^c^Indicate inconsistent fold change between RNAseq and qPCR

### Target prediction and network analysis

In our recent study, DE mRNAs were identified by mRNA sequencing analysis in the same breast muscle tissues of PeM and BPR [2]. To investigate potential interactions between miRNA and mRNA expression, miRDB (http://mirdb.org), an online tool for miRNA target prediction and functional annotations was used to predict target genes of validated, DE miRNAs. A total of 2194 genes (mRNAs) were predicted as potential targets for 8 qPCR validated DE miRNAs (except miR-126-3p which showed inconsistent fold change values between miRNAseq and qPCR) (data not shown). Target mRNAs for DE miRNA were integrated with our DE mRNA dataset (retrieved from Kong et al., 2017). Expressions showing opposite direction to corresponding miRNA (e.g., down-regulated transcripts that are targets of up-regulated miRNA in PeM) were chosen for further pathway analysis using the IPA. According to the miRNA-mRNA interaction criteria, 153 candidate target genes for 8 miRNAs were identified (Additional file 2) including 118 down-regulated transcripts potentially targeted by 7 up-regulated miRNAs (miR-2131-5p, miR-221-5p, miR-146b-5p, miR-10a-5p, let-7b, miR-125b-5p, and miR-146c-5p). Similarly, it was predicted that 35 up-regulated transcripts might be modulated by down-regulated miRNA (miR-206) in PeM muscle samples.

As results of pathway analyses with DE miRNA and their target genes, the top biological functions of target genes were identified by the IPA using its features, “Top Canonical Pathways” and “Physiological System Development and Function” (Table 3). The most relevant biological functions of DE miRNA and their target DE mRNA in skeletal muscle included axonal guidance signaling, glycine degradation, calcium signaling, serine biosynthesis, zymosterol biosynthesis, endocrine system development and function, embryonic development, organismal development, skeletal and muscular system development and function, and tissue organismal development.

**Table 3:** Top biological functions of target genes between PeM and BPR as presented by IPA

Top Canonical Pathways	*p*-value
Axonal Guidance Signaling	0.00991
Glycine Degradation (Creatine Biosynthesis)	0.0131
Calcium Signaling	0.0303
Serine Biosynthesis	0.0325
NRF2-mediated oxidative stress response	0.0361
Zymosterol Biosynthesis	0.0389
Physiological System Development and Function	*p*-value range
Endocrine System Development and Function	1.96E-02 - 2.56E-04
Embryonic Development	2.86E-02 - 4.33E-04
Organismal Development	2.92E-02 - 4.33E-04
Skeletal and Muscular System Developmental and Function	2.61E-02 - 6.36E-04
Tissue Development	2.86E-02 - 8.86E-04

## Discussion

In this study, an extensive set of miRNAs was identified by small RNA sequencing and their potential roles in muscle growth and feed efficiency were determined in PeM and BPR chickens. Among a total of 994 mature miRNAs identified (data not shown), the 38 mature miRNAs were abundantly present in breast muscle of PeM and BPR birds (Table 4). Of those, miR-1a-3p was the most abundant with average read per million reads (RPM) of 334,936.0 and 337,938.6 in BPR and PeM chickens, respectively (Table 4). A study conducted in mice showed the role of the elevated miR-1a-3p in suppressing multiple factors required for the phosphorylation of the c-Jun N-terminal kinases/Mitogen activated protein kinase (JNK/MAPK) pathway and thereby promoting the expression of transcriptional factor MyoD [17], a myogenic regulatory factor required for skeletal muscle development [18]. However, the role of miR-1a-3p in muscle growth in chickens has not been studied. The other miRNA, miR-133c-3p, that was abundantly expressed in chicken breast muscle [19], enhances skeletal muscle proliferation and differentiation by repressing SRF [11]. Similarly, miR-21 and miR-146c have been recently identified as miRNAs upregulated in chicken interdigital regression and associated with inflammation, cell senescence, and programed cell death [20]. Additionally, a previous study reported upregulated expression of miR-21 in breast muscle of low body weight chickens [9]. Other studies in chicken and rats have reported the inhibitory effect of miR-21 in proliferation of pre-adipocytes and renal tubular epithelial cells, respectively [21, 22]. In addition, miR-22-3p, miR-30a-5p, miR-30d, miR-10b, miR-148a, miR-146c-5p and miR-199 were also known as abundantly present in breast muscles [9]. Altogether, evidences in literature suggest that abundant miRNAs identified in our samples may play role in enhanced growth and development of breast muscle of modern broilers.

**Table 4:** Abundant mature miRNAs present in breast muscle of PeM and BPR chickens

	*RPM				*RPM		
miRNA	Ave. BPR	Ave. PeM	**Mean	miRNA	Ave. BPR	Ave. PeM	**Mean
gga-miR-1a-3p	334,936.0	337,938.6	336,437.3	gga-miR-133a-5p	6298.4	6064.0	6181.2
gga-miR-30c-5p	186,869.2	178,733.7	182,801.4	gga-miR-10b-5p	5047.2	8048.4	6547.8
gga-miR-126-3p	49,349.8	71,550.1	60,450.0	gga-miR-24-3p	5001.3	4388.1	4694.7
gga-miR-17-5p	58,039.9	53,308.0	55,673.9	gga-miR-16-5p	4105.2	5268.3	4686.8
gga-let-7b	26,643.5	31,809.5	29,226.5	gga-miR-301b-3p	4767.5	4666.8	4717.1
gga-miR-181a-5p	29,530.8	26,834.5	28,182.7	gga-miR-221-5p	2740.1	6499.7	4619.9
gga-miR-206	31,760.2	23,406.7	27,583.5	gga-miR-21-5p	3496.3	3803.9	3650.1
gga-let-7f-5p	19,774.2	16,839.1	18,306.6	gga-miR-20a-5p	3395.8	3528.7	3462.3
gga-let-7c-5p	15,467.4	16,304.0	15,885.7	gga-miR-451	4919.9	2846.0	3883.0
gga-miR-30d	16,441.3	15,214.6	15,827.9	gga-miR-10a-5p	2409.5	4459.7	3434.6
gga-miR-146c-5p	12,167.9	17,007.6	14,587.8	gga-miR-221-3p	2993.7	3519.9	3256.8
gga-miR-222a	10,032.7	12,212.6	11,122.6	gga-let-7 g-5p	3044.1	3207.3	3125.7
gga-miR-133c-3p	9874.8	11,030.0	10,452.4	gga-miR-103-3p	2819.7	2995.4	2907.6
gga-miR-30a-5p	9682.9	10,924.1	10,303.5	gga-miR-22-3p	1988.3	2714.4	2351.3
gga-miR-193a-3p	11,370.2	10,310.7	10,840.4	gga-miR-2954	1977.5	1520.8	1749.2
gga-miR-125b-5p	8184.5	12,684.1	10,434.3	gga-miR-1454	1545.4	1526.3	1535.9
gga-miR-26a-5p	8965.0	7300.4	8132.7	gga-miR-2131-5p	927.1	2505.3	1716.2
gga-miR-199-5p	8310.5	7656.8	7983.6	gga-miR-128-3p	1276.4	1466.2	1371.3
gga-miR-148a-3p	6703.5	6804.6	6754.1	gga-miR-146b-5p	713.6	1894.0	1303.8

*RPM denotes average Reads Per Million reads

**Mean is the average RPM value of BPR and PeM

Of eight DE miRNAs validated with qPCR, expressions of miR-146b-5p, miR-125b-5p, miR-2131-5p, let-7b, miR-221-5p miR-146c-5p, miR-126-3p, and miR-10a-5p were higher in PeM muscle while the miR-206 showed lowered expression in PeM. All DE miRNAs have been shown to be involved in muscle development in various animal species [5, 7, 9, 23–25]. MiR-206 is specifically expressed in skeletal muscle and functions in muscle differentiation and cell proliferation in chickens and human [26–28]. Let-7b has been reported to be abundantly expressed in breast and skeletal muscle in chickens and to play roles in growth regulation via targeting growth hormone receptor [29]. MiR-10a is a well characterized miRNA and is known to implicate with muscle development and myogenesis regulation in various animals [7, 23, 24, 30]. MiR-146b is a known regulator of skeletal myoblast differentiation in vitro and muscle regeneration in mice [25]. MiR-126 mediates vascular integrity and angiogenesis. It also elicits direct effects on regulation of skeletal muscle growth and activation of insulin like growth factor 1(IGF-1) [31, 32]. MiR-125b is known to regulate calcification of vascular smooth muscle cells. It targets IGF in both regenerating muscles and myoblasts [33]. There is evidence of expression of miR-221 controlled by Ras-MAPK pathway. It is involved in vascular smooth muscle proliferation and plays a role in progression from myoblasts to myocytes developing into fully differentiated phenotype [10, 34]. Therefore, all DE miRNAs identified in this study seem to be closely related with muscle growth and development in chickens to their mammalian counterparts.

**Fig. 2:**
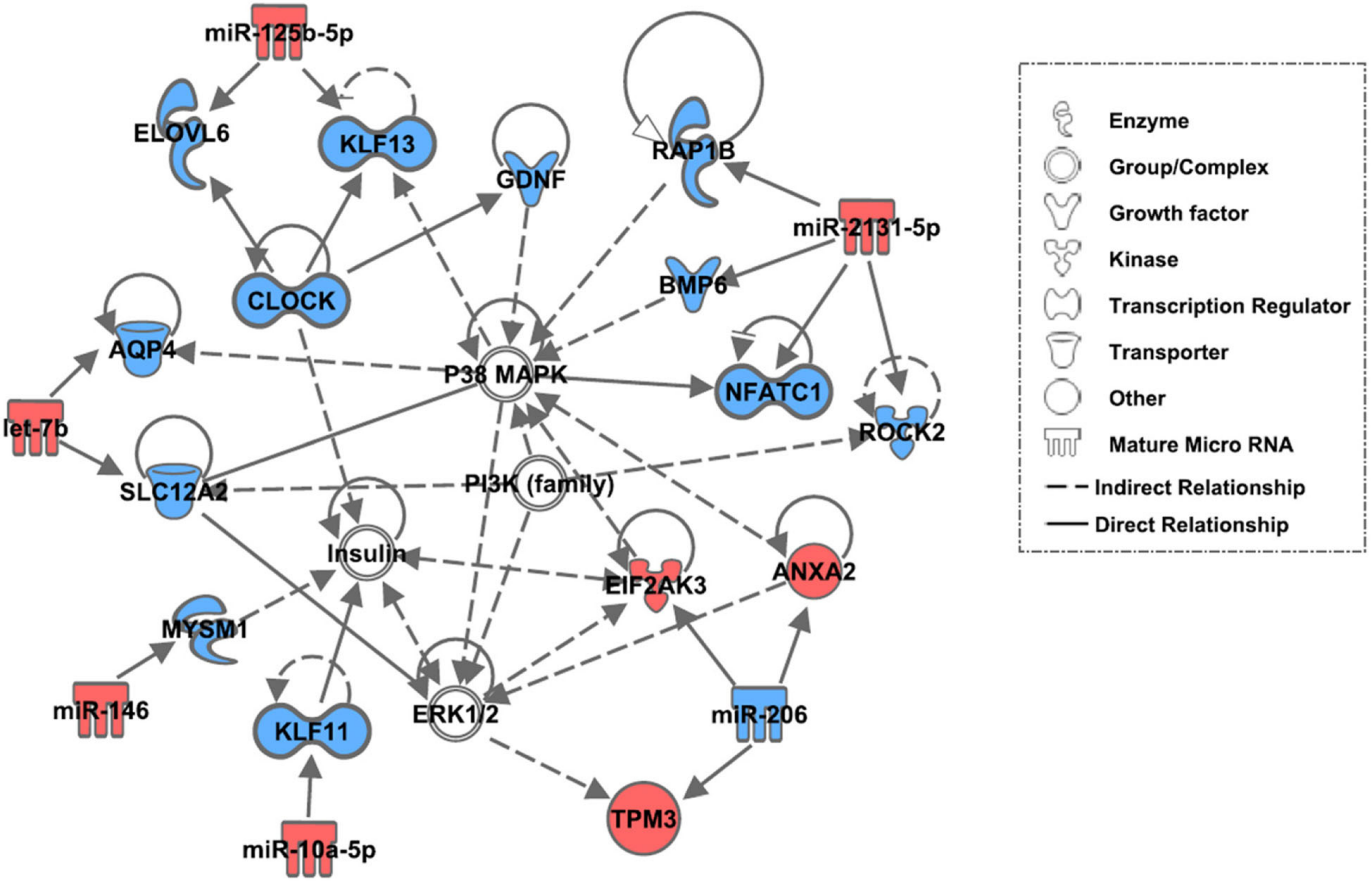
Network associated with P38 MAPK, ERK1/2, PI3K and insulin signaling pathways. Genes and miRNA filled with red are up-regulated while symbols in blue color are down-regulated in PeM in comparison to BPR

To understand cellular and physiological mechanisms in chickens muscle development, DE miRNA and their target DE mRNA were subjected to in silico pathway analysis using the IPA. The network analysis of DE miRNA-DE mRNA pairs (showing opposite expression patterns between miRNA and DE mRNA) were shown to be interlinked with P38 MAPK, ERK1/2, PI3K, and insulin-signaling pathways (Fig. 2). Further, functions associated with these genes include embryonic development, organ development, organismal development, and molecular transport. In the network, the up-regulation of annexin A2 (ANXA2), tropomyosin 3 (TPM3) and eukaryotic translation initiation factor 2 alpha kinase 3 (EIF2AK3) genes seemed to be directly regulated by down-regulation of miR-206 and their close association with P38 MAPK, ERK1/2, PI3K and insulin signaling cascades. ANXA2, lipocortin II or p36, which is a 36-kDa Ca^2+^-dependent protein of the annexin superfamily plays regulatory functions in proliferation, migration and cytoskeletal formation in muscle cells [35, 36]. TPM3 binds to actin filaments in muscle cells. In association with troponin complex, TPM3 has central role in controlling contraction of striated muscle in vertebrates [37]. EIF2AK3, a metabolic stress sensing protein kinase, phosphorylates eukaryotic translation initiation factor 2 and is also involved in controlling mitochondrial morphology and function [38, 39]. Therefore, it is possible that the interaction between miR-206 and its target genes may reflect the rapid myogenesis shown in breast muscle of PeM chickens. Interestingly, the down-regulated target genes of miR-146, miR-10a-5p, miR-125b-5p, miR-2131-5p and let-7b are also assigned to these signaling pathways.

**Fig. 3:**
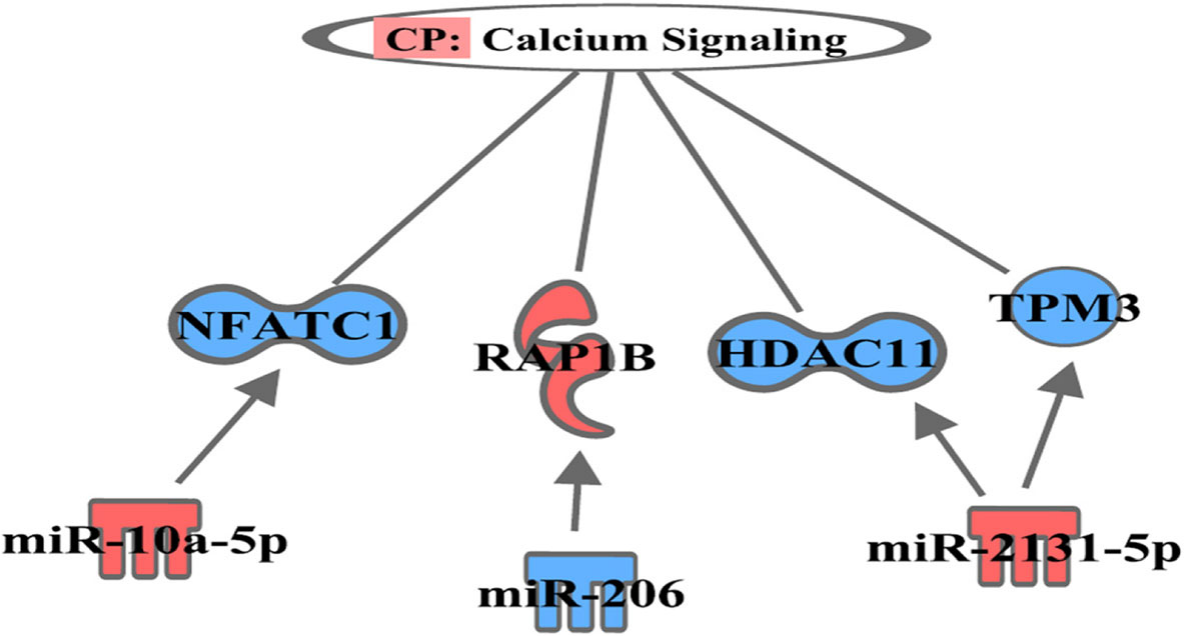
Pathway of calcium signaling predicted by IPA. Color symbols were indicated in the legend of Fig. 2

**Fig. 4:**
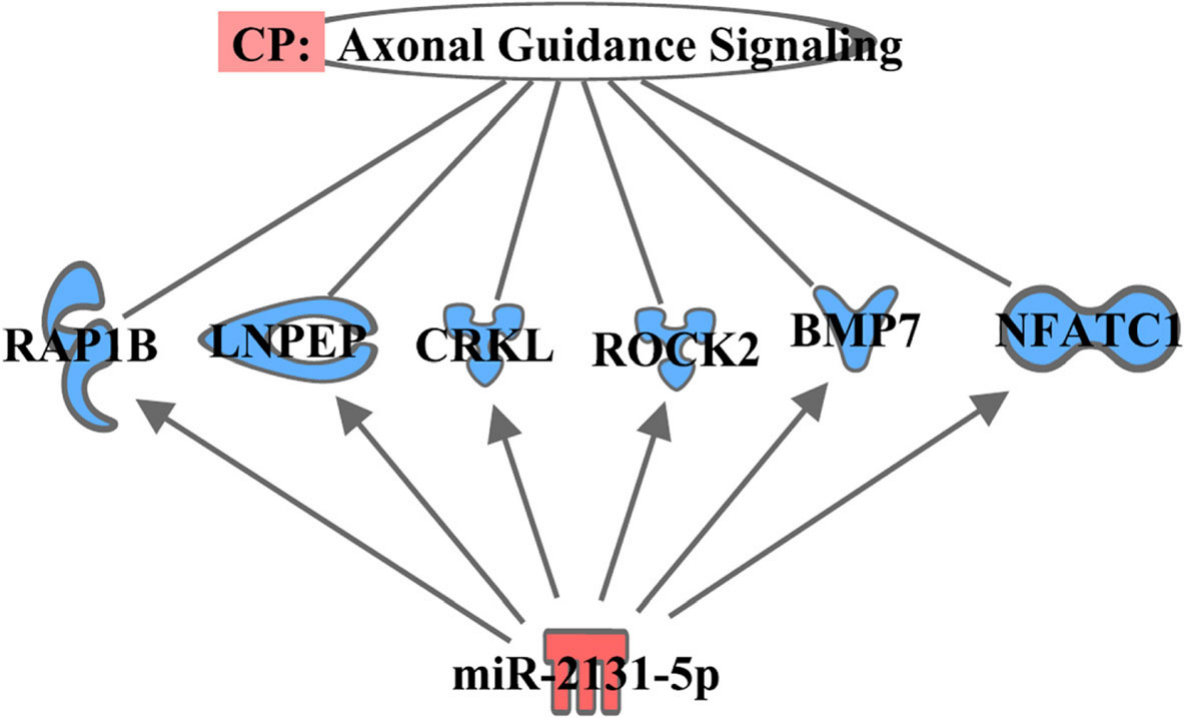
Pathway of axonal guidance. Color symbols were indicated in the legend of Fig. 2

**Fig. 5:**
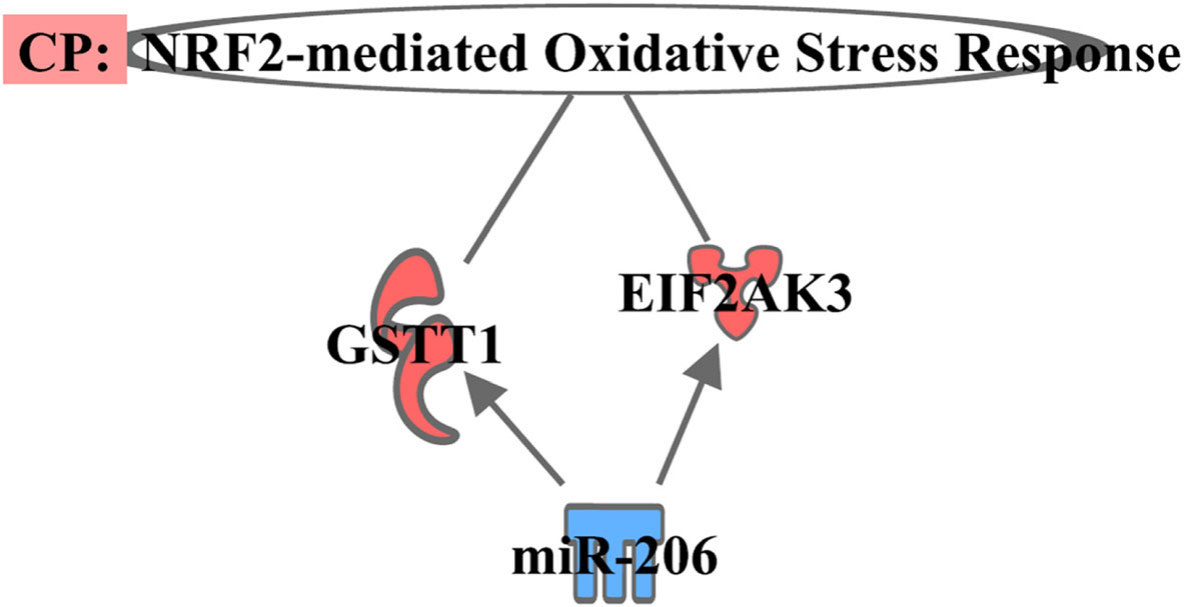
Pathway of NRF2-mediated oxidative stress response. Color symbols were indicated in the legend of Fig. 2

From the IPA canonical pathway analysis, we found that the targeted DE mRNAs were associated with calcium signaling (*p*-value 3.03E-02; Fig. 3), axonal guidance signaling (p-value 9.91E-03; Fig. 4), and NRF2-mediated oxidative stress response (p-value 3.61E-02; Fig. 5) pathways. The target genes involved in calcium signaling pathway include: HDAC11 gene (target of miR-10a-5p); a member of RAS oncogene family (RAP1B) and nuclear factor of activated T-cells 1 (NFATC1) genes (targets of miR-2131-5p); and TPM3 and bone morphogenetic protein 6 genes (BMP6) (targets of miR-206). It is well established that the homeostasis of intracellular calcium level is important for muscle growth and development. The increased intracellular level of Ca^2+^ can occur due to both poor Ca^2+^-ATPase activity and disturbance of sarcolemma integrity which results in hypercontraction of myofibers and degeneration of muscle mass [40–43]; the IPA suggests that this pathway might have been modulated by miR-10a-5p and miR-2131-5p thereby leading to larger muscle mass observed in PeM compared to BPR chickens. We also found the involvement of target genes of miR-2131-5p in axonal guidance signaling pathway; the predicted target mRNAs of miR-2131-5p included RAP1B, leucyl and cystinyl aminopeptidase, CRK like proto-oncogene, adapter protein, Rho associated coiled-coil containing protein kinase 2, BMP7, and NFATC1. Interestingly, Mutryn et al. (2015) recently suggested possible roles of axonal guidance signaling pathway in the breast muscle myopathy in chickens [40]. In a different study, miR-2131-5p has been reported as a mature miRNA specific to the avian lineage with unknown functions [44]. Hence, it is postulated that DE miR-2131-5p can play roles in regulation of axonal guidance signaling pathway in breast muscle of chickens. Further investigations and validation works might be warranted with regards to the implications of calcium metabolism and axonal guidance signaling in PeM samples and responsible physiological roles of DE miRNAs therein.

Last, we identified EIF2AK3 and glutathione S-transferase theta 1 (GSTT1) genes (targets of miR-206) involved in NRF2-mediated oxidative stress response pathway. NRF2 is one of the main factors responding to both oxidative and xenobiotic stresses. It plays a critical role in neutralizing oxidative stress by activating the expression of antioxidants and detoxifying enzymes [45]. The activation of EIF2AK3 and GSTT1 genes is associated with decreased level of reactive oxygen species [46, 47]. Previously, the augmentation of the NRF2-mediated oxidative stress response pathway is observed in breast muscle of higher feed efficient and rapidly growing chickens [48]. Therefore, increased expression of EIF2AK3 and GSTT1 genes, as a result of down-regulation of miR-206, implied the activation of canonical NRF2-mediated response pathway for scavenging reactive oxygen species from breast muscle of rapidly growing PeM chickens.

## Conclusion

Using miRNA sequencing, integrated analyses of miRNA-mRNA data and IPA, we were able to identify breast muscle specific miRNAs and their target genes whose concerted actions can contribute to rapid growth and higher feed efficiency in modern broiler chickens. We believe our comprehensive analysis enables us to better understand miRNA and their physiological roles in breast muscle growth in chickens. Future validation studies are warranted in regards to interactions between miRNA and target genes (e.g., in vitro transfection studies) to characterize functions of miRNAs and their specific targets in the context of rapid muscle growth and development.

## Additional files

Additional file 1: Read Per Million values of abundantly expressed miRNAs for indivial samples. (XLSX 22 kb)

Additional file 2: Target mRNAs of differentially expressed and qPCR validated miRNAs in PeM and BPR comparison. (XLSX 20 kb)

## Abbreviations

ANXA2: Annexin A2
ATP: Adenosine triphosphate
BMP7: Bone morphogenetic protein 7 gene
BPR: Barred Plymouth Rock
cDNA: Complementary DNA
Ct: Cycle threshold
DE: Differentially expressed
EIF2AK3: Eukaryotic translation initiation factor 2 alpha kinase 3
ERK1/2: Extracellular signal-regulated kinase
GSTT1: Glutathione S-transferase theta 1
HDAC: Histone deacetylase
IACUC: Institutional Animal Care and Use Protocol
IGF-1: Insulin like growth factor 1
IPA: Ingenuity Pathway Analysis
JNK: c-Jun N-terminal kinases
MAPK: Mitogen activated protein kinase
miRNA: MicroRNA
mRNA: messenger RNA
NFATC1: Nuclear factor of activated T-cells 1
NRF2: Nuclear related factor 2
PeM: Pedigree Male
PI3K: Phosphoinositide 3-kinase
qPCR: quantitative reverse transcription polymerase chain reaction
RAP1B: RAS oncogene family
SRF: Serum response factor
TPM3: Tropomyosin 3
